# Effect of respiratory motion on cardiac defect contrast in myocardial perfusion SPECT: a physical phantom study

**DOI:** 10.1007/s12149-019-01335-y

**Published:** 2019-01-24

**Authors:** Matti J. Kortelainen, Tuomas M. Koivumäki, Marko J. Vauhkonen, Mikko A. Hakulinen

**Affiliations:** 1 Department of Applied Physics, University of Eastern Finland, Kuopio, Finland; 2 Diagnostic Imaging Center, Kuopio University Hospital, Kuopio, Finland; 3 Department of Radiation Therapy, Central Finland Central Hospital, Jyväskylä, Finland

**Keywords:** SPECT, Myocardial perfusion imaging, Respiratory motion, Phantom

## Abstract

**Objective:**

Correction for respiratory motion in myocardial perfusion imaging requires sorting of emission data into respiratory windows where the intra-window motion is assumed to be negligible. However, it is unclear how much intra-window motion is acceptable. The aim of this study was to determine an optimal value of intra-window residual motion.

**Methods:**

A custom-designed cardiac phantom was created and imaged with a standard dual-detector SPECT/CT system using Tc-99m as the radionuclide. Projection images were generated from the list-mode data simulating respiratory motion blur of several magnitudes from 0 (stationary phantom) to 20 mm. Cardiac defect contrasts in six anatomically different locations, as well as myocardial perfusion of apex, anterior, inferior, septal and lateral walls, were measured at each motion magnitude. Stationary phantom data were compared to motion-blurred data. Two physicians viewed the images and evaluated differences in cardiac defect visibility and myocardial perfusion.

**Results:**

Significant associations were observed between myocardial perfusion in the anterior and inferior walls and respiratory motion. Defect contrasts were found to decline as a function of motion, but the magnitude of the decline depended on the location and shape of the defect. Defects located near the cardiac apex lost contrast more rapidly than those located on the anterior, inferior, septal and lateral wall. The contrast decreased by less than 5% at every location when the motion magnitude was 2 mm or less. According to a visual evaluation, there were differences in myocardial perfusion if the magnitude of the motion was greater than 1 mm, and there were differences in the visibility of the cardiac defect if the magnitude of the motion was greater than 9 mm.

**Conclusions:**

Intra-window respiratory motion should be limited to 2 mm to effectively correct for respiratory motion blur in myocardial perfusion SPECT.

## Introduction

Respiration is known to be responsible for visible artifacts in myocardial emission tomography images [1, 2] and interfere with the detectability of perfusion defects [3]. During the last two decades, respiratory-induced cardiac motion compensation has been investigated by several research groups with either positron emission tomography (PET) or single-photon emission computed tomography (SPECT) by applying a variety of methods. One common feature of all these methods is the recording of a signal that represents respiratory motion during image acquisition. This signal can be generated either directly from the emission data [4] or from motion tracking devices [5]. This signal is used either prospectively or retrospectively to sort emission data into respiratory windows [6]. One simple, straightforward approach is to only use emission data from an individual respiratory window to reconstruct an image with minimized respiratory motion [2, 7–14]. The obvious disadvantage of these respiratory gating methods, analogous to cardiac gating, is that since the image is produced with fewer emission photons than the non-gated image, the signal-to-noise ratio (SNR) is lower.

More advanced respiratory motion compensation methods utilize all of the acquired emission photons by co-registering the myocardial activity from all respiratory windows at some point in the reconstruction. For example, in the method of Kovalski et al., the respiratory-gated projection images are shifted relative to each other before summing them to reconstruct a motion-compensated image [15]. Another method is to reconstruct each respiratory window separately, co-register these images and compute their average as a motion-compensated image [16–20]. One other method involves estimating the inter-window motion from individually reconstructed windows and then modifying the observation matrix to finally reconstruct a motion-compensated image [4, 16, 21–23]. Alternatively, one can simultaneously reconstruct the image and estimate the cardiac displacement vectors by combining motion estimation and reconstruction into one optimization problem [24, 25].

In all of the above-mentioned methods, it is assumed that there is negligible intra-window residual respiratory motion. However, there is no clear consensus on how large the intra-window motion can be and still be acceptable: the number of applied respiratory windows in previous studies has varied from as few as three [10] to as many as sixteen [19]. Adopting a larger intra-window motion would make it possible to use fewer respiratory windows to cover the whole cardiac displacement between inspiration and expiration. This, in turn, would facilitate the process of inter-window motion estimation due to the higher SNRs in the individual respiratory windows and make the motion-compensated reconstructions computationally faster.

In the patient study of Dawood et al., the authors sought to determine the optimal number of respiratory windows by measuring the cardiac displacement using different numbers of windows and concluded that it would be preferable to use eight amplitude windows [26]. Their criterion was to detect the cardiac displacement up to a certain accuracy. Nonetheless, one important aim of myocardial perfusion tomography is to locate reliably perfusion defects in the myocardium, and this may be compromised by respiratory motion [3]. Therefore, in this present work, we have adopted a different approach and investigated the effect of respiratory motion on the cardiac defect contrast. The investigation was carried out by imaging a physical cardiac phantom and simulating respiratory motion during the list-mode data binning. The cardiac defect contrasts for several different magnitudes of motion in the physiologically relevant range of respiratory motion are reported. Our aim was to determine an optimal value for the intra-window residual motion for the individual respiratory windows.

## Methods

### Cardiac phantom

**Fig. 1:**
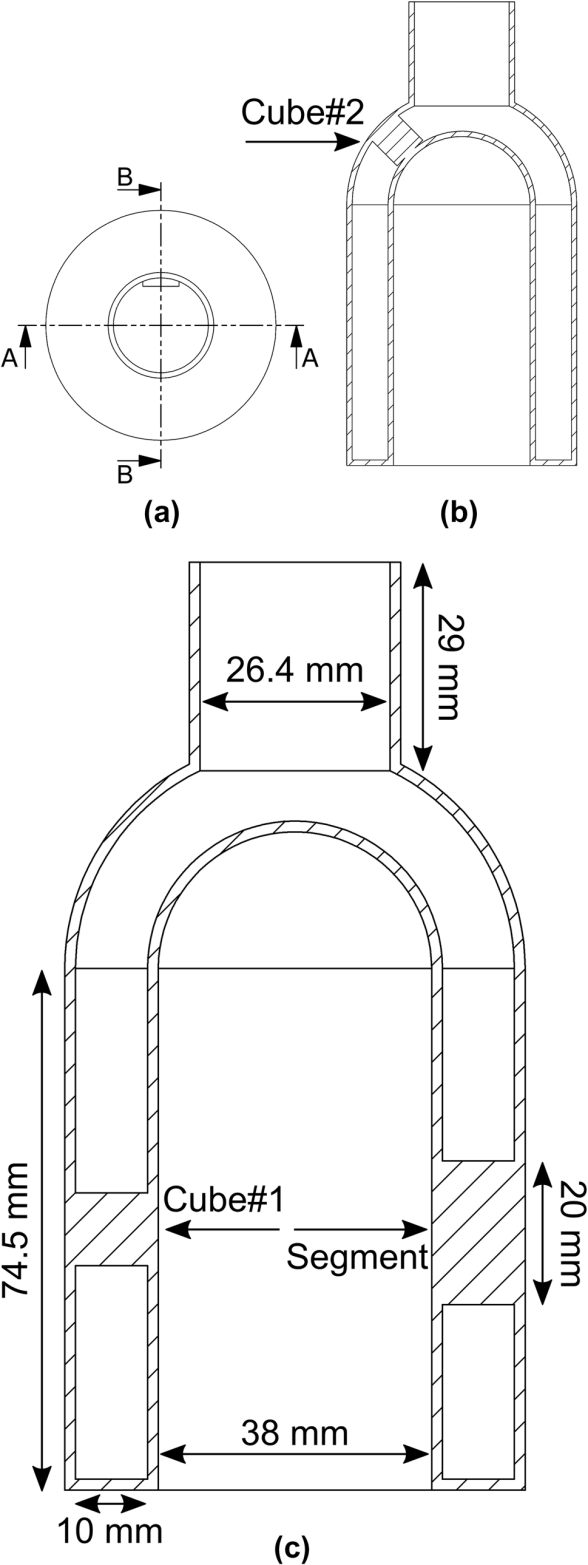
Blueprint of Kuopio Cardiac Phantom with 10-mm-thick defects. Top view (**a**). Section B**–**B (**b**). Section A**–**A through the middle of the phantom showing the dimensions (**c**). The side length of Cube#1 and Cube#2 is 10 mm

For the purposes of this work, a custom-designed cardiac phantom modeling the left ventricle of the heart, termed Kuopio Cardiac Phantom (KCP), was created (Fig. 1). KCP has a cylindrical–hemispherical shape; this was chosen to simplify the design and because this kind of shape is also applied in the left ventricular wall segmentation strategy applied in Emory Cardiac Toolbox (Emory University) [27]. On the top of the hemispherical apex, there is a cylindrical bottleneck to permit filling and emptying of the myocardial compartment of the phantom. This bottleneck can be closed with a rubber plug. Cardiac dimensions, such as myocardial wall thickness, were chosen to match human male anatomy [28].

KCP was made by additive manufacturing (3D printing) via powder bed fusion and using polyamide as the material [29]. Polyamide was chosen because in terms of radiodensity, it was found to be nearly soft tissue equivalent [200–300 Hounsfield units (HU)]. KCP was printed in two parts; these parts were coated with cyanoacrylate adhesive before gluing them together to prevent fluid absorption into the porous structure of the phantom [29]. KCP was found to remain waterproof even after over 8 h of testing.

KCP contains three artificial perfusion defects in its myocardial compartment: two cube-shaped defects, denoted Cube#1 and Cube#2, and one 20 mm high, 60° arc segment-shaped defect, denoted Segment. Cube#1 is located vertically in the middle of the cylindrical wall at 0° azimuthal angle, Segment is located vertically in the middle of the cylindrical wall at 180° azimuthal angle and Cube#2 is located in the hemispherical apex at 90° azimuthal and 45° polar angle (Fig. 1). In the design, the cubical defect shape was chosen for ease of implementation and the segmental defect shape was chosen to enable comparison with a commercially available cardiac phantom (Cardiac Insert™; Data Spectrum Corporation, NC, USA).

The defects were created by extending the 3D printing region from the epicardial wall towards the endocardial wall. The dimensions of the defects could be altered in the design before manufacturing: the side length of the cubes could be altered, as well as the thickness of the Segment [29]. In this study, a phantom with 10-mm-thick defects was produced.

### “Torso” phantom

**Fig. 2:**
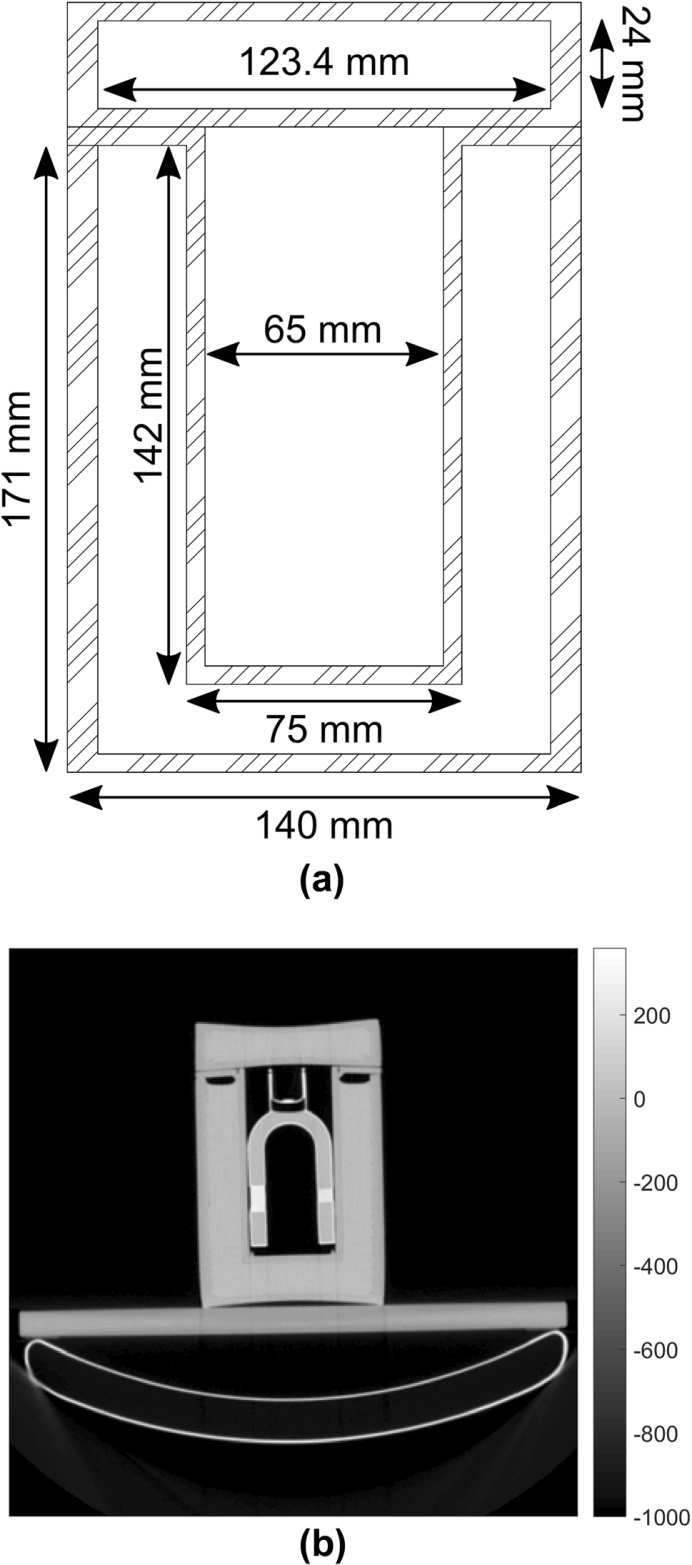
Blueprint of the cylindrical “torso” phantom. Cross-section through the middle of the phantom showing the dimensions (**a**). CT image of the water-filled “torso” phantom showing the water-filled cardiac phantom inside the “torso” phantom (**b**)

To mimic extra cardiac attenuation and scattering effects, a custom-designed “torso” phantom was created (Fig. 2). The “torso” was made from plastic that was found to be soft tissue equivalent in terms of radiodensity (−100–0 HU). It consists of three cylindrical parts: a large water tank, a small water tank and a holder for KCP. These parts could be stacked such that the holder part was submerged into the large water tank, forming a cylindrical cavity for KCP, surrounding it with a soft tissue-equivalent material (Fig. 2b).

### Image acquisition

In the clinical protocol applied in Kuopio University Hospital (Finland), the standard injected activity of Tc-99m for 1-day stress/rest myocardial perfusion imaging examination is 300 MBq at stress and 700 MBq at rest. Since it has been reported that approximately 1.7% of injected activity can be taken up by the myocardium [30], there would be approximately 14.6 MBq of Tc-99m in the myocardium at the time of rest image acquisition. To simulate clinical rest acquisition in this work and also to create several image reconstructions from the same list-mode data, KCP was filled with water and 121 MBq of Tc-99m.

Image acquisitions were performed with a dual-detector SPECT/CT system (Precedence; Royal Philips N.V., Netherlands). KCP was placed inside the “torso” phantom, which was placed on a plastic plate on top of the patient table to provide an even support surface for the phantoms (Fig. 2b). CT image acquisition was performed using the standard clinical protocol used in myocardial perfusion SPECT/CT studies (scan mode: helical, tube potential: 140 kV, exposure: 20 mAs/slice, pixel size: 1.171875 mm, matrix size: 512 × 512 and slice thickness: 4.267 pixels).

SPECT list-mode image acquisition was performed using a 90° detector configuration, 64 projection angles, acquisition time of 30 s per projection angle, low-energy high-resolution collimators (hole length 32.8 mm and diameter 1.4 mm) and a noncircular orbit from the right anterior oblique to the left posterior oblique angle. The orbit was chosen to match the distances between the detector and the cardiac phantom used in vivo during the acquisition (approximately 8.4 cm in the anterior view and 19.5 cm in the left view). An energy window of 140 keV ± 10% was used in the acquisition.

The phantoms were imaged twice, with two different KCP orientations. In the first orientation, KCP was positioned such that the line determined by midpoints of Segment and Cube#1 was parallel to the axis of rotation of the gamma camera. In this orientation, the Segment was situated in what would be the anterior cardiac wall, Cube#1 was situated in the inferior cardiac wall and Cube#2 was situated on the septal side of the hemispherical apex. We denote this orientation as orientation A (Fig. 4). In the second orientation, KCP was positioned such that the line determined by midpoints of Segment and Cube#1 was perpendicular to the axis of rotation of the gamma camera. In this orientation, the Segment was situated in what would be the septal cardiac wall, Cube#1 was situated in the lateral cardiac wall and Cube#2 was situated on the inferior side of the apex. We denote this orientation as orientation B (Fig. 4). SPECT image acquisitions of orientations A and B were started 41 and 117 min post-injection, respectively.

The orientations were chosen to investigate the respiratory motion effect on the defect contrast when these defects are located in different positions of the heart. Since motion blur was generated in the axial direction (see next subsection), in orientation A, the Segment and Cube#1 were located on the walls perpendicular to the direction of motion. In orientation B, the Segment and Cube#1 were located on the walls parallel to the direction of motion. In addition, we were able to investigate the defect contrast in two different apical cardiac positions (Cube#2).

### Generation of motion-blurred data

The list-mode data were processed into projection images of matrix size 128 × 128 and pixel size of 4.664 mm using custom-made scripts in the MATLAB R2015b environment (The MathWorks, Inc., MA, USA). In this work, instead of acquiring images of the moving phantom, motion blur was generated in the images during the list-mode data binning. A one-dimensional, uniform motion pattern was assumed along the rotation axis (*z*-axis) of the gamma camera.

Let *f(z)* denote a function

$$f\left( z \right)=~\left\{ {\begin{array}{*{20}{l}} {\frac{1}{P}~,~\quad \left| z \right| \leq ~\frac{P}{2}} \\ {0~,~\quad {\text{otherwise}},} \end{array}} \right.$$

where *P* is the full width of one pixel, and let *g*(*z*) denote the motion-blurring function

$$g\left( z \right)=~\left\{ {\begin{array}{*{20}{l}} {\frac{1}{M}~,~\quad \left| z \right| \leq ~\frac{M}{2}} \\ {0~,\quad {\text{~otherwise}},} \end{array}} \right.$$

where *M* is the full width of the motion blur. The motion-blurred function *h*(*z*) is then a convolution of function *f*(*z*) and motion-blurring function *g*(*z*)

$$\begin{aligned} h\left( z \right) & = ~f\left( z \right)*g(z) \\ &= ~\frac{1}{M}\frac{1}{P}\left\{ {r\left( {z+\frac{M}{2}+\frac{P}{2}} \right) - r\left( {z+\frac{M}{2} - \frac{P}{2}} \right) - r\left( {z - \frac{M}{2}+\frac{P}{2}} \right)+r\left( {z - \frac{M}{2} - \frac{P}{2}} \right)} \right\}, \\ \end{aligned}$$

where *r*(*x*) is the ramp function

$$r\left( x \right)=~\left\{ {\begin{array}{*{20}{l}} {x,\quad ~x \geq ~0} \\ {0,~\quad {\text{otherwise}}.} \end{array}} \right.$$

Since the motion blur spreads the activity to neighboring pixels on the *z*-axis, the following equation must hold,

$$1=~\mathop \smallint \limits_{{ - P/2}}^{{P/2}} f\left( z \right){\text{d}}z=~\mathop \sum \limits_{{i=~ - N}}^{N} {\xi _i},$$

where

$${\xi _i}=~\mathop \smallint \limits_{{\left( {2i - 1} \right)P/2}}^{{\left( {2i+1} \right)P/2}} h\left( z \right){\text{d}}z.$$

That is, the contribution *ξ*_*i*_ of the motion-blurred object’s pixel to the neighboring pixel *i* is computed as an integral of *h*(*z*) over the domain of pixel *i* and 2*N* + 1 is the number of neighboring pixels. With the above notations, we created motion-blurred projection images as follows:

1. For all *i*, generate a projection image from the list-mode data with a duration of *ξ*_*i*_*T*, where *T* is the acquisition time of one realization of the projection image.
2. Shift the projection image by *i* pixels in the axial direction of the camera.
3. Sum the shifted projection images.

**Fig. 3:**
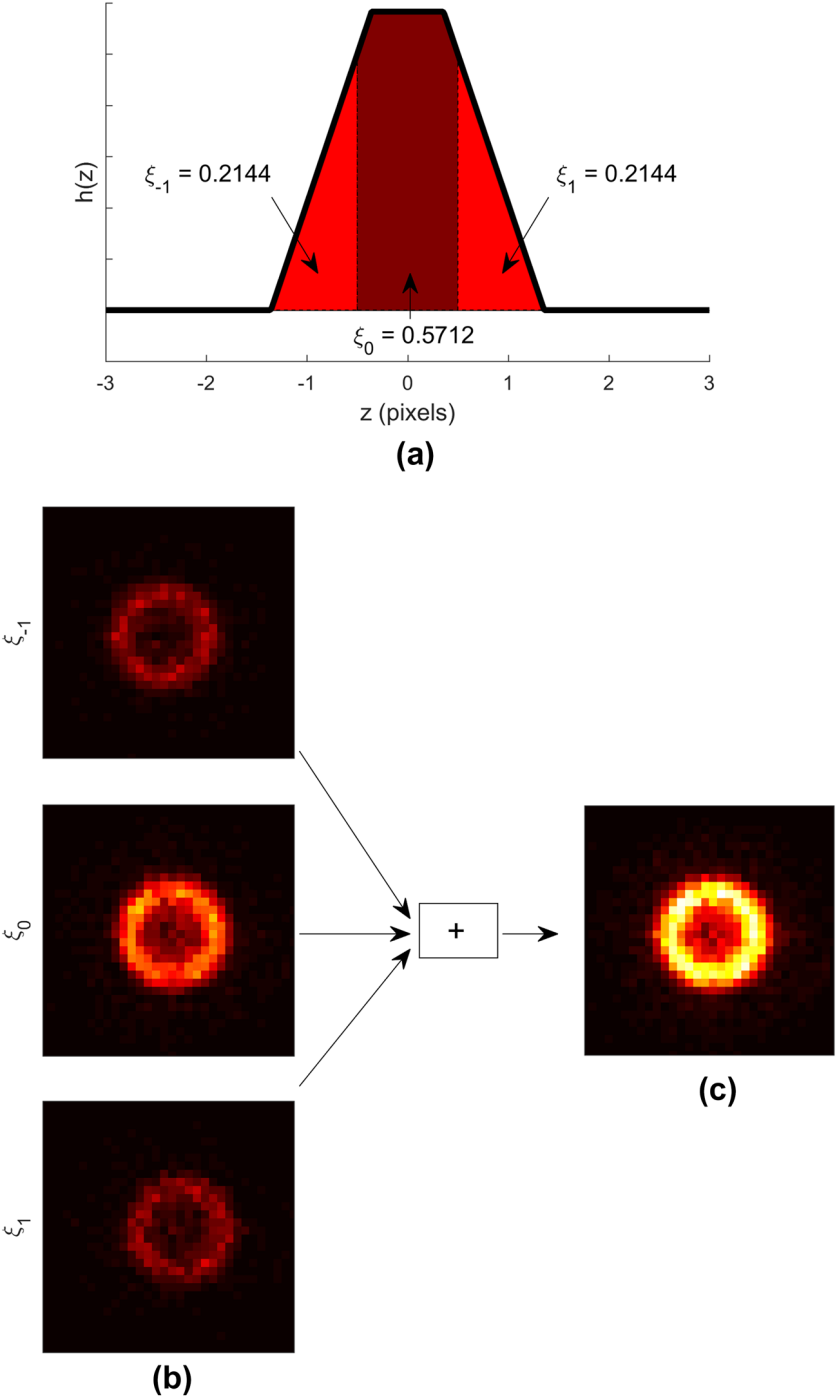
An example of generating a motion-blurred projection image. First, the contributions *ξ*_*i*_ to adjacent pixels due to motion are computed as integrals of the motion-blurred object function *h*(*z*). **a** Then, projection images with duration *ξ*_*i*_*T* are generated from the list-mode data and shifted by *i* pixels in the axial direction (right–left in the paper). **b** Finally, the shifted projection images are summed to yield a motion-blurred projection image. **c** In this example, the motion blur full width *M* is 8/4.664 pixels

An example of generating motion-blurred projection image is displayed in Fig. 3. In total, we generated projection images with motion blur full widths of 0 (stationary images), 1, 2, 3, 4, 5, 6, 7, 8, 9, 10, 12, 14, 16, 18 and 20 mm. These values were chosen because the heart has been reported to move by as much as 20 mm between expiration and inspiration [31]. For the remainder of this paper, we will call motion blur full width as motion magnitude for brevity.

To gather samples for statistical analysis, for each motion magnitude, we generated five different realizations from mutually non-overlapping sequences of list-mode data. For orientation A, the acquisition time of individual projection angle for each realization was *T* = 3.923 s and for orientation B, to compensate for decay, the acquisition time of individual projection angle for each realization was *T* = 4.542 s. The acquisition times were computed such that the product of injected activity at the start of the image acquisition and the acquisition time corresponded to the clinical situation (14.6 MBq and 30 s). For each realization, each motion-blurred image was generated from the same sequence of list-mode data as the stationary image.

### Image reconstruction

In the image reconstructions, we adopted a rotation-based approach. Rotation matrices were implemented using Gaussian interpolation [32]. Geometric collimator-detector blurring was modeled as distance-dependent Gaussian function [33]. CT images were converted to µ-maps using piecewise linear conversion [34]. These µ-maps were resampled to the SPECT matrix size and registered with stationary SPECT estimates using the normalized gradient fields method [35] with linear interpolation. Only three-dimensional translations were considered in the registration. Attenuation factors were computed using the central-ray approximation [36]. Correction for scattering was omitted. It is worth noting that while the attenuation-corrected reconstructions were made using motion-blurred projection images, the CT images were obtained from stationary phantoms and were not motion-blurred retrospectively. This corresponds to the clinical protocol in use in Kuopio University Hospital with the CT image being acquired during breath-hold.

For the reconstruction algorithm, we chose the ordered subsets expectation maximization (OSEM) that maximizes data log-likelihood [37]. We divided the projection data into eight subsets and ran the algorithm for ten iterations. A total of 80 updates has been found to be sufficient to maximize the contrast in Tc-99m images [38], and it is currently recommended in clinical practice for use in Hybrid Recon–Cardiology reconstruction software (HERMES Medical Solutions, Sweden). Reconstructions were carried out in the MATLAB R2015b environment (The MathWorks, Inc., MA, USA) using custom-made scripts.

### Perfusion analysis

To examine the well-known artificial decrease of myocardial uptake due to motion in anterior and inferior walls [1, 2], images of orientation B were analyzed with Carimas software v.2.9.4.0 (Turku PET Centre, Turku, Finland) [39]. The images were arranged into one dynamic image series (16 motion magnitudes and 5 realizations → 80 frames in total) to enable similar segmentation setup for all images. Using the standard 17-segment polar map division [40], anterior wall perfusion was computed as the average value of the anterior wall segments (basal-, mid-, and apical anterior segments), and inferior wall perfusion was computed as the average value of the inferior wall segments (basal-, mid-, and apical inferior segments). In addition, perfusion was examined in apex, lateral and septal walls. Septal wall perfusion was computed as the average value of apical septal, mid anteroseptal, mid inferoseptal, basal anteroseptal and basal inferoseptal segments, and lateral wall perfusion was computed as the average value of apical lateral, mid anterolateral, mid inferolateral, basal anterolateral and basal inferolateral segments.

### Contrast analysis

**Fig. 4:**
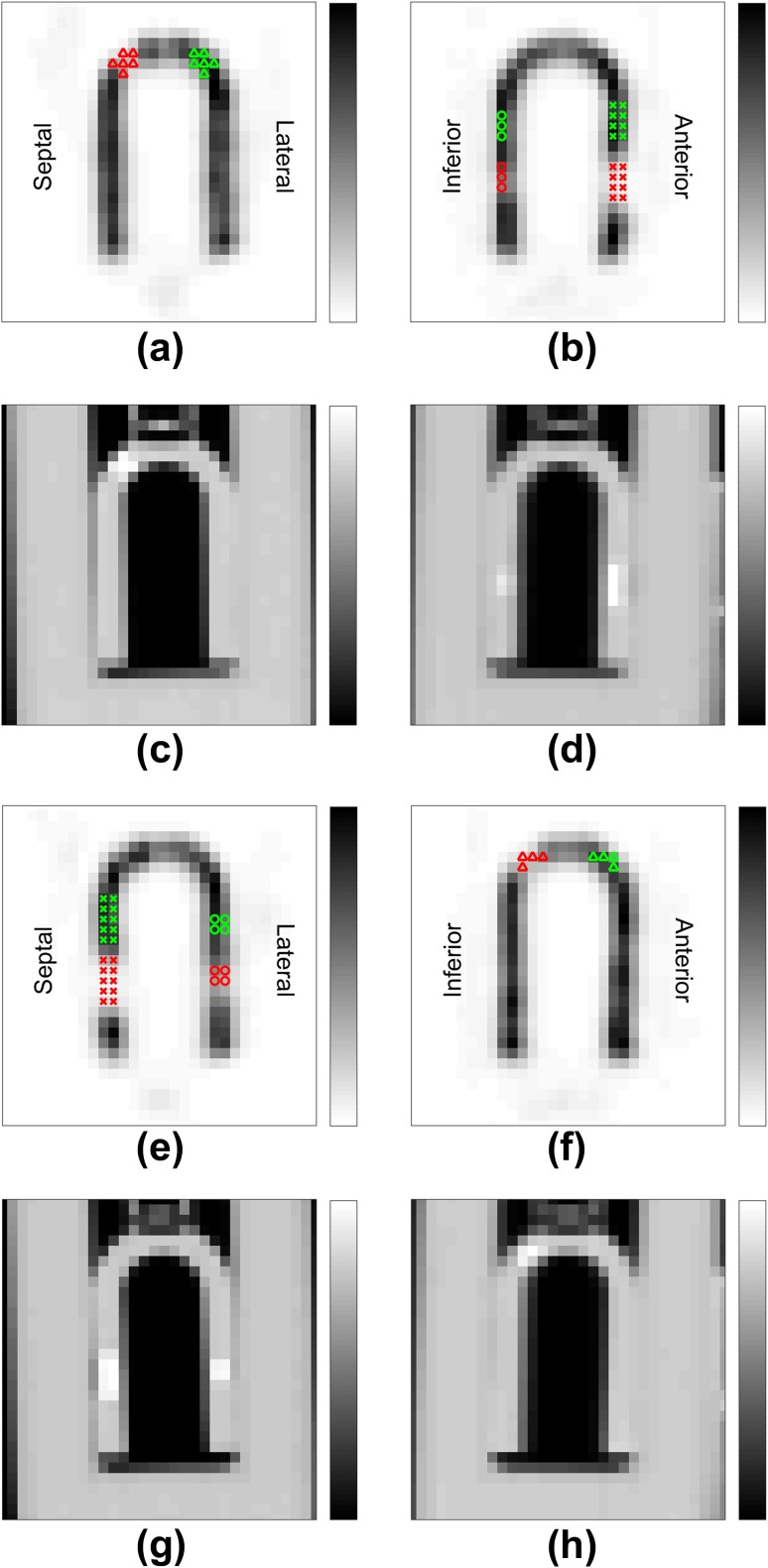
Definition of regions of interest (ROIs) in phantom orientations A (**a**–**d**) and B (**e**–**h**). Transverse views (**a, e**) and sagittal views (**b, f**) through the centers of SPECT images, and corresponding CT images (**c, d, g, h**). Defect regions are displayed with red and reference regions with green. Segment ROIs (×), Cube#1 ROIs (o) and Cube#2 ROIs (∆) are displayed

To assess the contrast between the cardiac defects and normal myocardium, we defined six regions of interest (ROIs) in both KCP orientations: three for the defects and three for their normal references. The ROIs were defined according to the CT images. As the defect regions had higher HU values than water, we defined the defect regions by increasing the threshold HU value until only a few clusters of voxels remained in the image. An HU value of 50 was chosen as the threshold for identifying defect region and the defect-containing clusters were found by considering the volumes of the clusters. Coordinates of the cluster voxels were recorded for further calculations. For Cube#1 and Segment, the reference regions were chosen as the regions adjacent (on the side of the apex) to their respective defect regions, and for Cube#2, the reference region was chosen symmetrically on the opposite side of the phantom (Fig. 4). All reference regions were of the same size and shape as their corresponding defect regions.

Once the ROIs were defined on the CT images, the reconstructed SPECT images were aligned with the CT images using the translation vectors computed previously in the CT/SPECT registration. Linear interpolation was used in the alignment. Using the defined ROIs on aligned SPECT images, the contrast *C* between defect and normal myocardium was computed as

$$C=~\frac{{{A_{{\text{normal}}}} - {A_{{\text{defect}}}}}}{{{A_{{\text{normal}}}}}},$$

where *A*_defect_ and *A*_normal_ are the average voxel values on defect ROI and normal myocardium ROI, respectively.

### Statistical tests

Statistical tests were performed in SPSS Statistics v.23 (IBM Corporation, NY, USA). Statistical significance of differences between motion-blurred images and stationary images was examined with a linear mixed model analysis. In the model, motion was treated as a fixed effect and the ordinal of realization as a random effect. Pairwise comparisons were performed between the motion-blurred images and the stationary images. Bonferroni adjustment was applied for multiple comparisons. *P* value of < 0.05 was considered statistically significant.

### Visual assessment

**Fig. 5:**
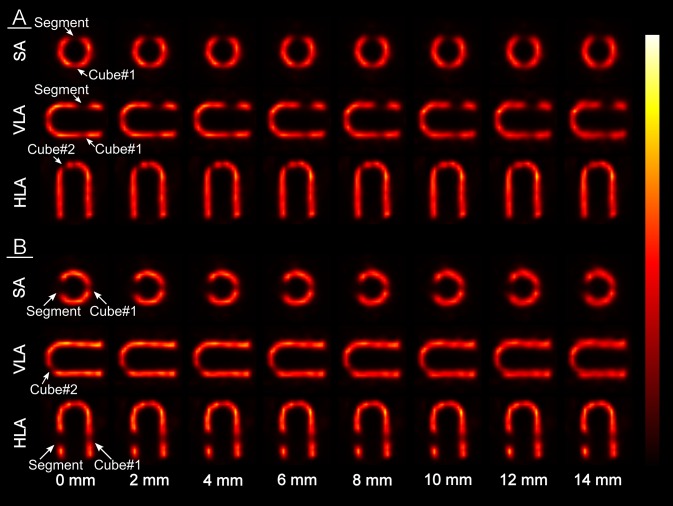
Short axis (SA), vertical long axis (VLA) and horizontal long axis (HLA) views of Kuopio Cardiac Phantom at orientations A and B at selected motion magnitudes. The cardiac defects are displayed with white arrows

To evaluate the differences between motion-blurred and stationary images visually, two experienced nuclear medicine physicians were recruited to assess the images in terms of defect visibility and perfusion (Fig. 5). Using the Quantitative Perfusion SPECT (QPS) 2012 program (Cedars-Sinai Medical Center, Los Angeles, CA, USA), the physicians were simultaneously shown two images—a stationary image and a motion-blurred image—but they were not aware of the extent of the motion magnitude in the motion-blurred image. The order of motion-blurred images was randomized. The physicians were asked to evaluate whether there were differences in the visibility of the cardiac defects or myocardial perfusion using a scoring system of 0–4 (0 = no difference, 1 = very small difference, 2 = small difference, 3 = medium-sized difference, 4 = large difference). The physicians scored the images as a consensus. The physicians viewed one realization of each motion magnitude in both phantom orientations, thus they viewed a total of 30 images.

## Results

Anterior and inferior wall perfusions were statistically significantly (*P* < 0.05) lower than in the stationary case at every motion magnitude (Table 1). Septal and lateral wall perfusions were statistically significantly (*P* < 0.05) lower than in the stationary case when the motion magnitude was 3 mm or greater (Table 1). Perfusion of the apex was statistically non-significantly (p > 0.05) different from the stationary case at every motion magnitude (Table 1).

**Table 1:** Average myocardial counts in phantom orientation B

Motion (mm)	Anterior wall (cts)	Inferior wall (cts)	Septal wall (cts)	Lateral wall (cts)	Apex (cts)
0	14,640 ± 190	13,779 ± 113	12,039 ± 110	12,473 ± 123	10,615 ± 191
1	14,519 ± 176*	13,666 ± 123*	12,035 ± 104	12,451 ± 117	10,636 ± 194
2	14,394 ± 177*	13,554 ± 120*	12,019 ± 105	12,433 ± 109	10,601 ± 203
3	14,270 ± 177*	13,433 ± 120*	12,006 ± 105*	12,411 ± 105*	10,625 ± 233
4	14,144 ± 167*	13,327 ± 116*	12,001 ± 101*	12,389 ± 93*	10,611 ± 246
5	14,018 ± 177*	13,210 ± 130*	11,998 ± 109*	12,363 ± 97*	10,662 ± 252
6	13,898 ± 173*	13,105 ± 126*	11,977 ± 105*	12,343 ± 100*	10,613 ± 204
7	13,782 ± 167*	12,982 ± 112*	11,977 ± 102*	12,324 ± 93*	10,625 ± 252
8	13,660 ± 168*	12,871 ± 108*	11,972 ± 113*	12,304 ± 91*	10,637 ± 241
9	13,528 ± 185*	12,766 ± 113*	11,952 ± 114*	12,282 ± 92*	10,621 ± 242
10	13,390 ± 189*	12,652 ± 111*	11,941 ± 119*	12,262 ± 92*	10,638 ± 186
12	13,072 ± 192*	12,339 ± 105*	11,899 ± 111*	12,201 ± 99*	10,641 ± 182
14	12,680 ± 187*	11,949 ± 92*	11,843 ± 113*	12,123 ± 86*	10,681 ± 185
16	12,241 ± 181*	11,515 ± 95*	11,789 ± 103*	12,031 ± 80*	10,654 ± 112
18	11,764 ± 184*	11,089 ± 95*	11,713 ± 106*	11,941 ± 77*	10,575 ± 126
20	11,290 ± 207*	10,611 ± 107*	11,627 ± 106*	11,830 ± 86*	10,578 ± 156

The results are expressed as mean ± standard deviation

*Denotes a statistically significant difference (*P* < 0.05) compared to the reference (0 mm)

In phantom orientation A, the contrast of Cube#2 was statistically significantly (*P* < 0.05) lower than that of the stationary case when the motion magnitude was 2 mm or greater, the contrast of Segment was statistically significantly (*P* < 0.05) lower as compared to the stationary case when the motion magnitude was 3 mm or greater, and the contrast of Cube#1 was statistically significantly (*P* < 0.05) lower as compared to the stationary case when the motion magnitude was 6 mm or greater, except at a motion magnitude of 8 mm (Table 2).

**Table 2:** Defect/normal myocardium contrast for orientation A

Motion (mm)	Segment	Cube#1	Cube#2
0	0.707 ± 0.017	0.408 ± 0.031	0.437 ± 0.011
1	0.705 ± 0.016	0.400 ± 0.037	0.428 ± 0.015
2	0.702 ± 0.015	0.398 ± 0.040	0.422 ± 0.016*
3	0.697 ± 0.017*	0.385 ± 0.037	0.414 ± 0.021*
4	0.694 ± 0.017*	0.390 ± 0.033	0.411 ± 0.017*
5	0.689 ± 0.016*	0.389 ± 0.034	0.406 ± 0.014*
6	0.684 ± 0.019*	0.377 ± 0.040*	0.395 ± 0.021*
7	0.678 ± 0.017*	0.377 ± 0.035*	0.388 ± 0.019*
8	0.674 ± 0.016*	0.383 ± 0.029	0.385 ± 0.023*
9	0.668 ± 0.016*	0.375 ± 0.024*	0.376 ± 0.018*
10	0.665 ± 0.018*	0.378 ± 0.018*	0.370 ± 0.013*
12	0.653 ± 0.019*	0.378 ± 0.024*	0.354 ± 0.022*
14	0.642 ± 0.019*	0.380 ± 0.042*	0.347 ± 0.019*
16	0.630 ± 0.021*	0.371 ± 0.044*	0.328 ± 0.026*
18	0.616 ± 0.026*	0.372 ± 0.034*	0.316 ± 0.019*
20	0.602 ± 0.028*	0.367 ± 0.036*	0.295 ± 0.025*

The results are expressed as mean ± standard deviation

*Denotes a statistically significant difference (*P* < 0.05) compared to the reference (0 mm)

In phantom orientation B, the contrast of Segment was statistically significantly (*P* < 0.05) lower than the stationary case when the motion magnitude was 3 mm or greater, the contrast of Cube#2 was statistically significantly (*P* < 0.05) lower in comparison to the stationary case when the motion magnitude was 5 mm or greater, and the contrast of Cube#1 was statistically significantly (*P* < 0.05) lower than the stationary case when the motion magnitude was 8 mm or greater (Table 3).

**Table 3:** Defect/normal myocardium contrast for orientation B

Motion (mm)	Segment	Cube#1	Cube#2
0	0.691 ± 0.021	0.413 ± 0.045	0.524 ± 0.035
1	0.690 ± 0.020	0.409 ± 0.043	0.518 ± 0.033
2	0.690 ± 0.018	0.407 ± 0.045	0.511 ± 0.031
3	0.687 ± 0.018*	0.407 ± 0.048	0.507 ± 0.032
4	0.687 ± 0.017*	0.401 ± 0.050	0.501 ± 0.032
5	0.687 ± 0.018*	0.399 ± 0.048	0.495 ± 0.028*
6	0.686 ± 0.019*	0.396 ± 0.054	0.490 ± 0.029*
7	0.685 ± 0.019*	0.390 ± 0.057	0.484 ± 0.028*
8	0.684 ± 0.018*	0.387 ± 0.058*	0.481 ± 0.032*
9	0.682 ± 0.018*	0.388 ± 0.056*	0.481 ± 0.031*
10	0.680 ± 0.018*	0.385 ± 0.058*	0.474 ± 0.030*
12	0.677 ± 0.018*	0.381 ± 0.054*	0.462 ± 0.030*
14	0.672 ± 0.018*	0.362 ± 0.054*	0.456 ± 0.034*
16	0.667 ± 0.018*	0.347 ± 0.054*	0.440 ± 0.033*
18	0.663 ± 0.017*	0.331 ± 0.058*	0.424 ± 0.027*
20	0.657 ± 0.019*	0.311 ± 0.056*	0.409 ± 0.024*

The results are expressed as mean ± standard deviation

*Denotes a statistically significant difference (*P* < 0.05) compared to the reference (0 mm)

In the visual assessment, differences between motion-blurred and stationary images were discernible in terms of perfusion if the motion magnitude was larger than 1 mm with orientation A and larger than 3 mm with orientation B (Table 4). In terms of defect visibility, the differences were discernible if the motion magnitude was larger than 10 mm with orientation A and larger than 9 mm with orientation B (Table 4).

**Table 4:** Visual assessment scores for orientations A and B

Motion (mm)	Orientation A				Orientation B			
	Defect visibility				Defect visibility			
	Segment	Cube#1	Cube#2	Perfusion	Segment	Cube#1	Cube#2	Perfusion
1	0	0	0	0	0	0	0	0
2	0	0	0	1	0	0	0	0
3	0	0	0	1	0	0	0	0
4	0	0	0	2	0	0	0	1
5	0	0	0	1	0	0	0	1
6	0	0	0	2	0	0	0	1
7	0	0	0	2	0	0	0	2
8	0	0	0	2	0	0	0	2
9	0	0	0	2	0	0	0	2
10	0	0	0	2	0	1	0	2
12	0	2	2	3	0	1	0	3
14	2	3	2	3	0	2	2	4
16	3	4	3	4	0	3	2	4
18	3	4	3	4	1	3	2	4
20	3	4	4	4	2	3	4	4

Compared to 0 mm motion, the difference is: 0 = non-existent, 1 = very small, 2 = small, 3 = medium-sized, 4 = large

## Discussion

In this study, we investigated the effect of respiratory motion on myocardial perfusion and contrast of various cardiac defects by imaging a custom-made cardiac phantom and generating motion-blurred data during list-mode data processing. We found that a motion magnitude as small as 1 mm could significantly affect anterior and inferior wall perfusion (Table 1), and a motion magnitude as small as 2 mm could significantly affect defect contrast (Table 2). In addition, we found that the motion influenced the defect contrast differently depending on the shape and position of the defect.

Our perfusion analysis revealed significant associations between the motion magnitude and the anterior and inferior wall perfusions (Table 1). Septal and lateral wall perfusions also decreased as a function of motion but the decrease was slower (Table 1). The perfusion of the apex, on the other hand, remained statistically unchanged as motion increased (Table 1). In the simulation study conducted by Kovalski et al., axial cardiac motions of 6 and 12 mm induced up to 3–4% and 10–12% changes in polar map segments [15]. These results are quantitatively comparable to our values (approximately 5% reduction in myocardial counts at 6 mm and 10–11% at 12 mm in anterior and inferior walls). On the contrary, Pitman et al. found that the reduction in counts in the anterior and inferior walls is barely visible if the magnitude of respiratory motion is less than 10 mm [1]. This difference may be explained by the fact that they used a lower number of projection angles, a larger voxel size, a smaller number of iterations in the iterative reconstruction and low-pass filtering [1], which all contribute to lower spatial resolution and thus complicate the detection of small differences attributable to motion.

The decrease in the defect contrast as a function of motion depends on the position and shape of the defect. In orientation A, the contrast of Segment decreased more rapidly as a function of motion blur than the contrast of Cube#1 (Table 2), whereas it was the opposite in orientation B (Table 3). In the study of Yang et al., a channelized hotelling observer analysis found that the visibility of lateral wall defects was more strongly affected by motion than that of the defects in the anterior and inferior walls [3]. Based on our results, the same phenomenon was found for Cube#1 defects but not for Segment defects. This may be explained by the fact that for Segment, there was very little activity surrounding the defect in the direction of motion in both orientations. Therefore, in orientation A, as the average counts on the reference region (anterior wall) decreased due to motion blur, the contrast values decreased; however, in orientation B, the average counts in the reference region (septal wall) remained almost unchanged which led to a slower decrease in the contrast values.

The contrast of Cube#2 decreased more rapidly as a function of motion than either of the other two defects in both orientations. This indicates that defects in the apical region are more sensitive to respiratory motion than defects in the wall segments that are oriented either perpendicularly or tangentially to the direction of motion. As far as we are aware, the visibility of defects in the apical region in the presence of motion has not been evaluated before and therefore this is the first study to report this result.

The results of the visual assessment were slightly discordant with the quantitative results. While the physicians recognized very small differences in perfusion already at 2 mm motion magnitude (Table 4), it was more difficult for them to discern differences in defect visibility. Only at motion magnitudes of 10 mm or greater were the differences scored higher than as non-existent (Table 4). These results suggest that small changes in defect contrast are not visually perceivable as effectively as with ROI-based quantification, but small changes in myocardial perfusion are detectable.

This study had some limitations. First, the induced motion pattern was simplified. Real respiratory motion is a nonrigid transformation that can only be approximated as an axial rigid translation [31]. Second, the cardiac phantom was imaged in only one cranio-caudal angle, such that the long axis of the phantom was perpendicular to the rotational axis of the camera. Third, all the studied defects were solid defects. In real-life scenarios, a region with a perfusion defect will display at least some activity, unless it consists exclusively of scar tissue. However, despite these limitations, this study represents a useful benchmark for more advanced studies that will utilize more realistic phantoms and investigate more complex types of motion.

Our quantitative results showed that there is no clear “safe” respiratory motion magnitude which could be included in individual respiratory windows for motion correction. The defect contrast and anterior and inferior wall perfusion decreased continuously as a function of motion. However, our quantitative results help to estimate how much a particular motion magnitude will affect the results. For example, on average, a motion of 5 mm led to an approximately 7% reduction of contrast in apicoseptal defects (Table 2). Furthermore, a motion of 2 mm resulted in less than a 5% reduction of contrast at every location studied in this work (Tables 2, 3) and less than a 2% reduction of average myocardial counts in anterior and inferior walls (Table 1). In addition, a motion of 2 mm produced only a very small visually discernible difference in myocardial perfusion. For example, for a common respiratory motion of 12 mm, six respiratory windows should be used to effectively correct for respiratory motion blur in the images. Therefore, if possible, it is advisable to obtain a priori knowledge about the extent of respiratory motion to choose the correct number of respiratory windows.

## Conclusions

Defect contrast declines as a function of respiratory motion magnitude in the apical cardiac region more rapidly than on the cardiac walls which are perpendicular or parallel to the direction of motion. The intra-window respiratory motion should be limited to 2 mm per window to effectively correct for respiratory motion blur.
